# Reduced Thickness of the Retina in *de novo* Parkinson’s Disease Shows A Distinct Pattern, Different from Glaucoma

**DOI:** 10.3233/JPD-223481

**Published:** 2024-04-23

**Authors:** Asterios Chrysou, Tuomas Heikka, Sygrid van der Zee, Jeffrey M. Boertien, Nomdo M. Jansonius, Teus van Laar

**Affiliations:** a Department of Neurology, University of Groningen, University Medical Center Groningen, Groningen, The Netherlands; b Department of Ophthalmology, University of Groningen, University Medical Center Groningen, Groningen, The Netherlands

**Keywords:** Parkinson disease, *de novo*, glaucoma, amacrine cells, biomarkers, inner plexiform layer Registration: ClinicalTrials.gov Identifier: NCT04180865.

## Abstract

**Background:**

Parkinson’s disease (PD) patients experience visual symptoms and retinal degeneration. Studies using optical coherence tomography (OCT) have shown reduced thickness of the retina in PD, also a key characteristic of glaucoma.

**Objective:**

To identify the presence and pattern of retinal changes in *de novo*, treatment-naive PD patients compared to healthy controls (HC) and early primary open angle glaucoma (POAG) patients.

**Methods:**

Macular OCT data (10×10 mm) were collected from HC, PD, and early POAG patients, at the University Medical Center Groningen. Bayesian informative hypotheses statistical analyses were carried out comparing HC, PD-, and POAG patients, within each retinal cell layer.

**Results:**

In total 100 HC, 121 PD, and 78 POAG patients were included. We showed significant reduced thickness of the inner plexiform layer and retinal pigment epithelium in PD compared to HC. POAG patients presented with a significantly thinner retinal nerve fiber layer, ganglion cell layer, inner plexiform layer, outer plexiform layer, and outer photoreceptor and subretinal virtual space compared to PD. Only the outer segment layer and retinal pigment epithelium were significantly thinner in PD compared to POAG.

**Conclusions:**

*De novo* PD patients show reduced thickness of the retina compared to HC, especially of the inner plexiform layer, which differs significantly from POAG, showing a more extensive and widespread pattern of reduced thickness across layers. OCT is a useful tool to detect retinal changes in *de novo* PD, but its specificity versus other neurodegenerative disorders has to be established.

## INTRODUCTION

Parkinson’s disease (PD) is a progressive neurodegenerative disorder of the central nervous system, also affecting the retina [1]. Retinal thinning has aroused interest as a possible biomarker of the disease [2, 3]. Past work has established reduced thickness of the retina in patients with PD [1]; however, very few studies have been published on *de novo* treatment-naive PD cohorts [4, 5] and most studies focused on a subset of retinal layers. In order to understand how PD affects the retina, it is important to understand when and where the changes start.

Novel methods like spectral domain optical coherence tomography (SD-OCT) have renewed interest in the retinal changes over time in neurodegenerative disorders like PD. SD-OCT allows for a by-layer thickness assessment of the retina and previous research has shown reduced thickness of the retinal nerve fiber layer (RNFL), the ganglion cell layer (GCL), and the inner-plexiform layer (IPL) [1]. The IPL contains the amacrine cells, important modulator cells with a dopaminergic, GABA-ergic and cholinergic role [6]. As such, they could be of importance in the early detection of PD. In addition to these three layers, state-of-the-art analysis software allows for the assessment of seven other retinal layers.

Published SD-OCT studies have compared PD to glaucoma, a common degenerative eye disease, primarily affecting the retina and optic nerve. Both disorders present with retinal thinning, vision impairment, and have been reported to co-occur [1, 7, 8]. These studies reported statistically significant differences, existing of a thinner RNFL and ganglion cell complex (GCC; the combined RNFL, GCL, and IPL) in glaucoma, compared to PD patients and healthy controls (HC) [9, 10]. Data regarding retinal changes in PD patients over time were inconclusive, varying from no change to a quite significant retinal thinning within 2.5 years [11, 12]. Interpretation of these studies, however, is not straightforward due to variance in disease stages of the included patients, as well as differences in the types and dosages of medication used, including dopaminomimetics, which could modulate the risk of developing glaucoma [13].

## MATERIALS AND METHODS

### Study design

The primary aim of this study was to identify the presence and pattern of retinal changes in *de novo,* treatment-naive PD patients, compared to HC and early primary open angle glaucoma (POAG) patients. For this purpose, we recruited three age-matched groups: *de novo* treatment-naive PD patients, early POAG patients, and HC. An a-priori power analysis was conducted to calculate the sample size, which was reported in the DUPARC protocol [14]. The most recent meta-analysis on OCT imaging in PD reported overall mean effect sizes of 0.45 for several retinal cell layers, comparing HC to PD patients [1]. For a similar effect size, with an alpha of 0.05, and a two-tailed comparison of means, a sample size of 79 subjects for each group (PD and HC) would be sufficient to achieve a power of 0.80. No comparison is published in the literature comparing *de novo* PD patients and clinically defined early POAG patients. Therefore, a formal power analysis was not possible.

We assessed the retinal structure with SD-OCT and split the retina into 10 cell layers, reporting the IPL and GCL distinctly (commonly reported together as GCIPL), and compared retinal thicknesses across all three groups. As a secondary analysis, we calculated within the PD patients correlations between reduced thickness of the retina and disease severity and cognition. Lastly, we ran a machine learning random forest classifier to evaluate the informative utility of SD-OCT, using 10 cell layer data, in order to distinguish PD patients from HC, and PD- from POAG patients.

### Study population

The inclusion criteria for PD patients were: newly diagnosed (*de novo)*, with a disease duration <3 months, and treatment-naive. The diagnosis of PD was based on the Clinical Diagnostic Criteria of the Movement Disorder Society, made by a movement disorders specialist, and a confirmed dopaminergic striatal deficit on ^18^F-FDOPA PET scanning [15]. The study protocol was approved by the Medical Ethical Review Board of the University Medical Center Groningen (NL60540 DUPARC, clinicaltrials.gov: NCT04180865) [14]. All participants provided written informed consent. The study followed the Declaration of Helsinki.

PD patients with any ophthalmic disorder that may influence retinal thickness assessment with OCT were excluded. The clinical data of all baseline patients were evaluated by an ophthalmologist at intake. In total, 33% of patients were invited for reevaluation by a clinician. Only one of these patients was excluded due to an incident diagnosis of glaucoma. Details of the ophthalmic assessment are described in the data collection subsection. In case of abnormal test results, the tests were repeated and the findings were evaluated by an ophthalmologist. Out of 38 repeated tests (39%), mainly due to abnormal visual fields related to a learning effect, only one patient was diagnosed with POAG, and excluded thereafter. The POAG patients were selected from the Groningen Longitudinal Glaucoma Study (GLGS), which consists of glaucoma patients and glaucoma suspects, who presented at the department of ophthalmology of the UMCG. The GLGS study started in 2000 as an observational cohort study. A detailed description of the population and study goals of the GLGS has been published [16]. Glaucoma patients were diagnosed based on a reproducibly abnormal test result on standard automated perimetry (defined as ‘Glaucoma hemifield test outside normal limits’), compatible with glaucoma and without any other explanation, in at least one eye. We only included patients with a diagnosis of POAG. Patients with secondary glaucoma or conditions such as pseudoexfoliative- or pigment dispersion glaucoma, or angle closure glaucoma, were excluded. We focused on early POAG, with the visual field parameter ‘mean deviation’ not worse than –6 dB at the time of the SD-OCT. Each included eye had to meet these visual field criteria. The first available SD-OCT scan of each patient was used. The average mean deviation (standard deviation) was –3.7 (1.5) dB at scan time. We also excluded patients with comorbid neurological disease (e.g., cerebral infarction, cerebellar ataxia, epilepsy, meningioma) or any ophthalmic disorder affecting the retina, not related to glaucoma.

HC were recruited via the ophthalmology clinic of the UMCG (mainly spouses of patients), with the following exclusion criteria: known retinal disease, intraocular pressure ≥22 mmHg as measured with non-contact tonometry, a positive family history of glaucoma, or an abnormal visual field screening test as measured with Frequency Doubling Technology (C20-1 screening mode; Carl Zeiss Meditec, Dublin, CA, USA). Recruitment of HC took place between 2016–2020. HC scans with retinal abnormalities were excluded.

### Data collection

SD-OCT data were collected at the ophthalmology clinic of the UMCG (Canon HS-100 OCT, software version 4.1.0; Canon, Tokyo, Japan). A 10×10 mm macular scan was made, centered around the fovea. We focused on the inner retinal cells (retinal ganglion cells and amacrine cells). These cells are not in the fovea and the vast majority of them are located in a circular region with a diameter of 6 mm centered at the fovea (that is, within 3 mm eccentricity; [17]), which made us adopt a 6×6 mm region centered at the fovea as our region of interest (ROI). Because scan quality is pivotal for an accurate measurement of the retinal layer thicknesses, we verified the quality of the scans in three different ways [18]. First, SD-OCT scans were selected based on an automated software quality assessment (Canon OCT software, quality of ≥4). Second, all processed scans were judged by two persons (AC and TH) for B-scan gaps (due to eye movements or blinks) and segmentation errors. Finally, a quality check was done on the final thickness data, using the ‘identify unusual case’ function (SPSS 23, IBM, Armonk, NY, USA), followed by a closer look at identified outliers for erroneous segmentation, which resulted in the exclusion of three patients. No manual correction of segmentation took place.

In the PD- and POAG groups, visual fields were assessed monocularly with the Humphrey Field Analyzer 2 (Carl Zeiss Meditec, Dublin, CA, USA), using the 24-2 grid with SITA Fast testing algorithm. Furthermore, visual acuity in PD patients was assessed monocularly with a LogMAR chart after optimizing refraction for the viewing distance. Static contrast sensitivity was assessed monocularly with the Pelli-Robson Contrast Sensitivity Chart (Mason, OH, USA). Measurements were performed at 1 m with optimal correction for the viewing distance. Color vision was assessed binocularly with the Farnsworth Panel D-15, and the Lanthony Desaturated Panel D-15. Intraocular pressure was assessed with non-contact tonometry (NT-530, Nidek, Tokyo, Japan). Retests (if the initial intraocular pressure was above 22 mmHg) were performed with Goldmann applanation tonometry. Cognition was assessed using the Montreal Cognitive Assessment (MoCA).

### Data analysis

Finally, 121 PD-, 78 POAG patients, and 109 HC fulfilled the in-/exclusion criteria. We further excluded 9 HC, to improve the matching between the HC and PD group on age. A sub-analysis with Bayesian informative hypothesis evaluation (BAIN) Welch’s *t*-tests (see below), comparing retinal cell layer thickness in the PD group between both sexes, showed that gender had no effect on retinal thickness.

SD-OCT DICOM files were extracted from the Canon software. The scans were processed with the Iowa Reference Algorithms (Version 3.80; Retinal Image Analysis Lab, Iowa Institute for Biomedical Imaging, Iowa City, IA), using the 68 HVF grid, to segment the retina in the macular area into 10 layers [19–21]. Averages were calculated over the entire grid across each cell layer, including the following layers: retinal nerve fiber layer (RNFL), ganglion cell layer (GCL), inner plexiform layer (IPL), inner nuclear layer (INL), outer plexiform layer (OPL), outer nuclear layer (ONL), inner segment/outer segment junction (IS/OS), outer segment layer (OSL), outer segment to RPE junction (OPR), and retinal pigment epithelium (RPE). A sub-analysis was carried out to verify that there were no significant differences between the left and right eyes of patients. After this process, we pooled eye data of participants with both eye scans available, taking the average layer thickness of both eyes, per cell layer, within each participant. Participants with one good eye scan were included as such.

To compare retinal cell layers between the three groups, we used BAIN Welch’s *t*-tests [22–25]. This method allowed us to test two hypotheses simultaneously. We compared two groups at the same time within each cell layer, and produced Bayes factors that expressed the likelihood ratio of each hypothesis based on the data. The two hypotheses were “a”: the null H0 (equal group means) was tested versus H1 (first mean larger than second mean) and “b”: the null H0 (equal group means) versus H2 (first mean smaller than second mean). Bayes factors are always positive. A Bayes factor of one represents a lack of strong evidence. A number higher than one favored hypothesis “a”, and a number smaller than one favored hypothesis “b”. To identify the error and to evaluate any (un)certainty in selecting the most likely hypothesis, we multiplied the prior probabilities, which were established upfront (50% per hypothesis and 33.3% per possible outcome), by the likelihood produced by our data, yielding the posterior probabilities, the error probability per selected hypothesis. BF10: the likelihood ratio (0-∞) of each hypothesis of cell layer thickness. A likelihood ratio of one is equal likelihood, units above one or below one express increased/decreased likelihood with direction of first “more likely” and second: “less likely”. The hypotheses are represented by H0: Equal, H1: Bigger, and H2: Smaller. The posterior probability is the prior probability modified by the observed data, with probability being expressed as a number ranging from 0 to 100, with a higher number representing a higher probability. We also reported the means and 95% credible intervals for all cell layer measurements, which were used as an additional measure of uncertainty. The chosen method quantified the support for each hypothesis, without the need for correction of multiple comparisons. Welch’s *t*-test does not require equal variances, only normality. Before analysis, Q–Q plots were made to assess this. The demographic variables were also assessed for normality with Q–Q plots. We compared PD patients to HC, and also PD patients to POAG patients. No comparisons were made between POAG patients and HC. In this paper we report likelihood ratios of Bayes Factor 10 (BF10), which is the Bayes factor in favor of H1 over H0.

### Correlation analyses

Correlation analyses were carried out with clinical variables within the PD patients. To evaluate the correlation between the retina and disease severity, we correlated the thickness of the layer that differed most between PD patients and HC with the total UPDRS III score. To evaluate links between the retina and cognition, we correlated the thickness of the concerning layer with the MoCA score. We controlled for age as a possible confounder.

### Machine learning classification

To evaluate the predictive utility of SD-OCT, we used a machine learning random forest classification analysis, with balanced categories, comparing all 10 cell layers between PD patients and HC, and between PD and POAG patients. The 10 cell layers were included as predictors, whereas disease status was the target. First, we classified two groups, PD patients and HC. The data (*n* = 221 participants) was split into three parts: train *n* = 141, validate *n* = 36, and test *n* = 44. Secondly, we classified PD –and POAG patients. The data (*n* = 199 participants) was again split into three parts: train *n* = 128, validate *n* = 32, and test *n* = 39.

## RESULTS

Table 1 presents the characteristics of our study populations, including UPDRS III scores and Hoehn & Yahr, MoCA, age and sex, and number of participants.

**Table 1:** Characteristics of the study population

	Parkinson’s disease	Healthy controls	Primary open angle glaucoma
N	121	100	78
Age (y)	65.1 (8.7)	62.7 (6.5)	65.7 (9.7)
Sex M/F	87/34	46/54	33/45
UPDRS III	30.4 (11.2)
Hoehn &Yahr	2 (1–2)
MoCA	26 (24–27)

Non-normally distributed variables (Hoehn & Yahr, MoCA) reported as median (interquartile range, 25th–75th percentile), Sex reported as number of participants. MoCA, Montreal Cognitive Assessment; UPDRS, Unified Parkinson’s disease Rating Scale.

### PD retinal cell layer comparisons to HC and to POAG patients

The SD-OCT comparisons between PD patients and HC showed a significant and specific reduced thickness in the retina of PD patients. The mean thickness of the IPL in PD patients was thinner; 34.2 (4.1) μm in PD versus 36.1 (3.9) μm in HC, and BF10 was 72. The RPE was also thinner in PD patients; 14.0 (0.4) μm in PD versus 14.2 (0.4) μm in HC, with a BF10 of 83. The remaining cell layers were equal in thickness between PD and HC.

The SD-OCT comparisons between PD -and POAG patients showed a significant overall reduced thickness in the retina of POAG patients. The following layers were thinner in POAG- compared to PD patients: the RNFL, GCL, IPL, OPL, OPR and the IS/OS. The mean RNFL thickness was 28.0 (6.0) μm in POAG and 30.5 (5.2) μm in PD with a BF10 of 18.8. The GCL thickness was 26.4 (6.4) μm in POAG- versus 34.8 (5.6) μm in PD patients, whereas the BF10 was 4.6×10^18^. The IPL thickness was 30.0 (4.6) μm in POAG-, compared to 34.2 (4.1) μm in PD patients, with a BF10 of 1.6×10^8^. Furthermore, the OPL thickness in POAG patients was 20.9 (2.8) μm versus 22.3 (3.1) μm in PD patients, with a BF10 of 21.4. The OPR thickness in POAG patients was 17.7 (3.8) μm compared to 19.4 (3.6) μm in PD patients, with a BF10 of 12.3. Lastly, the IS/OS thickness in POAG patients was 9.6 (1.7) μm compared to 10.0 (1.4) μm in PD patients, with a BF10 of 0.9.

**Fig. 1:**
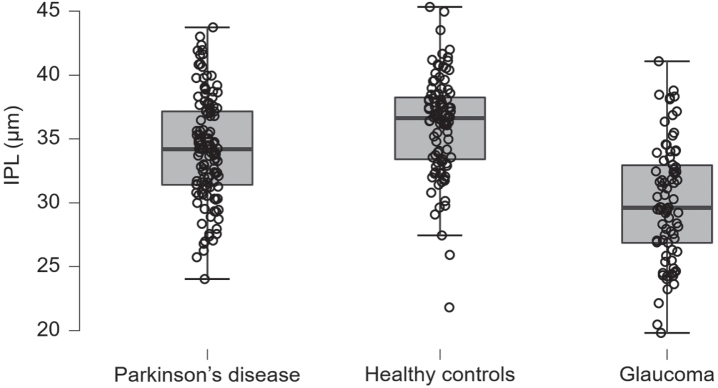
Inner plexiform layer. The inner plexiform layer thickness in Parkinson’s disease patients, healthy controls, and primary open angle glaucoma patients, units in microns, the whiskers represent the interquartile range. IPL, inner plexiform layer.

Only the RPE was thinner in PD patients with 14.0 (0.4) μm versus 14.2 (0.5) μm in POAG patients, with a BF10 of 0.02. The comparisons between the remaining cell layers of PD - and POAG patients were inconclusive. Table 2 presents the mean optical coherence tomography layer thickness data per group, displayed by cell layer. Table 3 presents the Welch’s *t*-test results of the optical coherence tomography group comparisons in Bayes factors and posterior probabilities. Figure 1 displays the IPL retinal thicknesses in PD patients, HC and POAG patients.

**Table 2:** Layer thicknesses (in μm) as assessed with optical coherence tomography for the three groups

	Parkinson’s disease	Healthy controls	Primary open angle glaucoma
RNFL	30.5 (5.2; 29.6–31.5)	31.0 (5.2; 30.0–32.0)	28.0 (6.0; 26.7–29.3)
GCL	34.8 (5.6; 33.8–35.8)	34.2 (4.6; 33.3–35.1)	26.4 (6.4; 25.0–27.8)
IPL	34.2 (4.1; 33.4–34.9)	36.1 (3.9; 35.3–36.9)	30.0 (4.6; 29.0–31.0)
INL	36.7 (4.0; 36.0–37.5)	35.9 (3.0; 35.3–36.5)	35.2 (6.2; 33.8–36.5)
OPL	22.3 (3.1; 21.7–22.8)	23.1 (2.8; 22.6–23.7)	20.9 (2.8; 20.3–21.5)
ONL	89.2 (6.9; 87.9–90.4)	89.7 (6.3; 88.4–90.9)	88.6 (8.2; 86.8–90.4)
ISOS	10.0 (1.4; 9.8–10.3)	10.4 (1.7; 10.0–10.7)	9.6 (1.7; 9.2–10.0)
OSL	16.2 (3.1; 15.6–16.7)	15.5 (2.9; 15.0–16.1)	17.5 (3.7; 16.7–18.3)
OPR	19.4 (3.6; 18.7–20.0)	18.9 (3.1; 18.3–19.5)	17.7 (3.8; 16.9–18.6)
RPE	14.0 (0.4; 13.9–14.1)	14.2 (0.4; 14.1–14.3)	14.2 (0.5; 14.1–14.3)

Retinal thicknesses by cell layer in microns. Data presented as mean (standard deviation; 95% credible interval). GCL, ganglion cell layer; INL, inner nuclear layer; IPL, inner plexiform layer; IS/OS, inner segment/outer segment junction; ONL, outer nuclear layer; OPL, outer plexiform layer; OPR, outer photoreceptor and subretinal virtual space; OSL, outer segment layer; RNFL, retinal nerve fiber layer; RPE, retinal pigment epithelium.

**Table 3:** Optical coherence tomography group comparisons

Parkinson’s disease	Healthy controls		Primary open angle glaucoma	
	Hypothesis	BF10	Posterior probability	BF10	Posterior probability
RNFL	H0: Equal	0.858	0.05
	H1: Bigger	0.122	0.104	0.017	0.001
	H2: Smaller	0.044	0.037	18.85	0.949
GCL	H0: Equal	0.836	0
	H1: Bigger	0.038	0.031	0.006	0
	H2: Smaller	0.159	0.133	4.562×10^18^	1
IPL	H0: Equal	0.014	0
	H1: Bigger	72.042	0.986	0.009	0
	H2: Smaller	0.014	0	1.635×10^8^	1
INL	H0: Equal	0.628	0.479
	H1: Bigger	0.025	0.016	0.026	0.012
	H2: Smaller	0.567	0.356	1.062	0.509
OPL	H0: Equal	0.401	0.045
	H1: Bigger	1.474	0.591	0.017	0.001
	H2: Smaller	0.021	0.008	21.442	0.955
ONL	H0: Equal	0.862	0.855
	H1: Bigger	0.115	0.099	0.051	0.044
	H2: Smaller	0.045	0.038	0.118	0.101
ISOS	H0: Equal	0.703	0.523
	H1: Bigger	0.394	0.277	0.026	0.014
	H2: Smaller	0.028	0.02	0.887	0.464
OSL	H0: Equal	0.683	0.186
	H1: Bigger	0.027	0.018	4.369	0.811
	H2: Smaller	0.437	0.299	0.02	0.004
OPR	H0: Equal	0.814	0.075
	H1: Bigger	0.035	0.028	0.018	0.001
	H2: Smaller	0.193	0.157	12.306	0.924
RPE	H0: Equal	0.012	0.216
	H1: Bigger	83.214	0.988	3.617	0.78
	H2: Smaller	0.014	0	0.021	0.005

GCL, ganglion cell layer; INL, inner nuclear layer; IPL, inner plexiform layer; IS/OS, inner segment/outer segment junction; ONL, outer nuclear layer; OPL, outer plexiform layer; OPR, outer photoreceptor and subretinal virtual space; OSL, outer segment layer; RNFL, retinal nerve fiber layer; RPE, retinal pigment epithelium. BF10: the likelihood ratio (0-∞) of each hypothesis of cell layer thickness. A likelihood ratio of one is equal likelihood, units above one or below one express increased/decreased likelihood with direction of first “more likely” and second: “less likely”. We compared, per cell layer, Parkinson’s disease patients to HC and Parkinson’s disease patients to POAG patients. The hypotheses are represented by H0: Equal, H1: Bigger, and H2: Smaller. The posterior probability is the prior probability modified by the observed data, with probability being expressed as a number ranging from 0 to 100, with a higher number representing a higher probability.

### Correlation analyses

The uncorrected correlation of the IPL thickness and total UPDRS III score was significant (*n* = 121, Pearson’s *r* = –0.19, *p* = 0.04). After having adjusted for age, the significance disappeared (Pearson’s *r* = –0.09, *p* = 0.32). The correlation of the IPL thickness and MoCA score was not significant (uncorrected: Pearson’s *r* = 0.02, *p* = 0.82; adjusted for age: *r* = –0.11, *p* = 0.23). The correlation of the joint GCL-IPL thickness and MoCA score, conducted to compare this data with the literature, proved to be not significant (uncorrected: Pearson’s *r* = 0.13, *p* = 0.15; adjusted for age: *r* = 0.07, *p* = 0.46). Lastly, the IPL thickness was not significantly correlated with the Hoehn & Yahr scores (Pearson’s *r* = –0.16, *p* = 0.08).

### 3.3 Machine learning classification

For PD patients compared with HC, 69 trees were grown and, after training and validation, the test accuracy was 0.57. An evaluation of variable importance showed the highest total increase in node purity for the IPL (0.013) and OPL (0.010). The area under the receiver operating characteristic curve (AUROC) was 0.71.

**Fig. 2:**
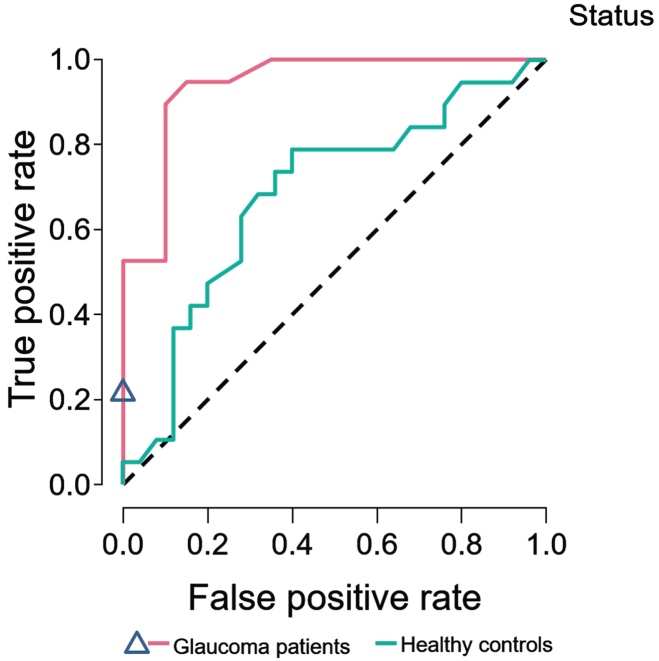
Random forest classifier. Random forest classifier, SD-OCT 10 cell layers. ROC curves of Parkinson’s disease patients predicted against healthy controls, and primary open angle glaucoma patients.

For PD- compared with POAG patients, 63 trees were grown. After training and validation, the test accuracy was 0.82. An evaluation of variable importance showed the highest total increase in node purity for the GCL (0.085) and RPE (0.046), with an AUROC of 0.92. Figure 2 shows the ROC curves for PD compared to HC and to POAG patients.

### Ophthalmic assessments

Visual acuity was normal and the refractive error was as expected in an elderly European population [26]. The contrast sensitivity showed a trend for reduction in PD patients (the mean of the contrast sensitivity distribution was close to the mean minus two standard deviations of the normative data [27]). The intraocular pressure was normal [28]. The average visual field mean deviation was significantly lower than the expected value of 0 dB (–1.7 dB (2.4; 95% credible interval: –2.1, –1.2), BAIN one sample *t*-test BF10 = 6.998×10^10^) [29]. The confusion index of the Farnsworth D-15 test was significantly larger compared to the reference population (1.3 (0.4), BAIN Welch’s *t*-test, BF10 = 1.017×10^6^), but did not reach the typical, much higher values for congenital color blindness (typically 2-3 for protanomaly and deuteranomaly; typically 4 for protanopia and deuteranopia) [30]. The selectivity index was also significantly different, with a larger mean compared to the reference population (1.6 (0.3), BF10 = 39.9). Angle was not significantly different compared to the reference population (56.7 (31.6), BF10 = 0.1). No normative data were available for the Lanthony (desaturated) D-15 test.

## DISCUSSION

We found a significantly reduced thickness of the IPL and RPE in PD patients compared to HC. This could reflect a loss of amacrine cell dendrites, possibly indicating a loss of amacrine cell bodies. The finding of a reduced thickness of the IPL in our PD cohort is in line with two previous publications on OCT in *de novo* PD populations, however these studies also found a reduced thickness of the RNFL in PD patients, which was not the case in our study [4, 5]. Despite being significantly different at a group level, the observed IPL thinning is small, making it less likely to become a future biomarker for PD. A possible explanation for the lack of RNFL thinning in our data may be the *de novo* disease stage of our PD patients. RNFL thinning possibly might occur later in the course of PD. Our POAG patients had significantly thinner retinas compared to PD, with a much more diffuse pathology, affecting several retinal cell layers. Only the OSL and RPE layers were thinner in PD. However, not only different layers were affected in PD compared to POAG patients, but also the magnitude of thinning differed significantly, with POAG patients showing a more extensive damage compared to PD patients. Previous studies have reported a joint GCL-IPL thinning in PD patients [1]. Most commercial SD-OCT devices do not provide segmentation software, being capable of separating these two layers. Therefore, most publications reported these layers together as GCL-IPL. Consequently, it is plausible that published studies may have underestimated the thinning of the IPL, and overestimated the effect size for the GCL. Our findings add a new perspective onto pre-existing published data, where the IPL layer has not been a subject of discussion. Similarly, it was not shown previously that the GCL is unaffected in PD patients, as shown by our data. Therefore, GCL and IPL should be analyzed separately in all future OCT studies. The GCL was especially affected in POAG, whereas in PD the IPL was the most affected layer. The IPL contains the neurotransmitters dopamine, GABA and acetylcholine, among others, which also play a pivotal role in PD [6]. The IPL is also linked to dopaminergic degeneration and alpha-synuclein aggregation [31]. Histopathological data showed that alpha-synuclein and Lewy neurites are present within axons and dendrites of the IPL, which might contribute to its reduced thickness [32]. The amacrine cells appear to be unaffected, but their synaptic connections projecting to the IPL are reduced by 60% [33]. Changes in the IPL thickness might be explained by a lack of dopaminergic input to the IPL. At the time of PD diagnosis generally 50% of the dopaminergic input from the substantia nigra to the striatum is lost [34]. This might also reflect the dopaminergic state of the retina. Recent work has provided evidence of retinal dopaminergic neuronal atrophy, with shorter and thicker dendrites, and a disturbed plexus [35]. The retinal dopamine cells also showed a significantly reduced (35%) amount of synaptic contacts with melanopsin-containing retinal ganglion cells [33]. This supports an important role of dopaminergic deficiency in the retina, possibly reflecting the loss of dopaminergic input in the striatum. Therefore, it is important to analyze retinal layers in PD without the influence of dopamino-mimetics.

We also found a significant RPE thinning in PD patients, whereas the absolute change in RPE thickness was relatively small. The etiology and meaning of that finding is yet unclear. The RPE has been coupled previously to the pathogenesis of PD. A conditioned medium of RPE cells was able to produce glial cell line-derived neurotrophic factor and brain-derived neurotrophic factor, which protects dopaminergic neurons against injury, and promotes the synthesis of dopamine [36]. So, reduced thickness of the RPE could be part of the overall neurodegeneration in PD, reflecting a loss of neuroprotective mechanisms.

Previous OCT-Angiography studies have shown foveal avascular zone capillary remodeling [37, 38]. PD patients present with thinner superficial and deep vascular plexuses compared to HC, and parafoveal thinning [37, 38]. Furthermore, an inverse association has been reported between the size of the foveal avascular zone and foveal thinning in PD [38]. However, a meta-analysis of OCT-Angiography studies did not replicate these results, reporting no significant differences between PD and HC in the foveal avascular zone, and the foveal and parafoveal superficial plexuses [39]. In contrast, the whole superficial vascular plexus was found to be thinner in PD compared to HC. The authors advised for a careful interpretation of these results, due to limited high-quality data in the literature, and the impact of test-retest variability on OCT-Angiography measurements, thereby suggesting a need for higher statistical power [39, 40]. We did not investigate the fovea independently in this study, as we focused primarily on the inner retinal cells, which are absent in the fovea. The inner retinal cells are present in the parafovea; however we did not do an independent analysis of this area. Rather, we focused on the whole macular area (6×6 mm), of which the parafovea represents a small part. We based our focus on (1) our previous meta-analysis, where the entire macula was thinned in PD, and the fovea least affected [1], (2) the fact that the vast majority of the inner retinal cells are located in this region [17], and (3) the observation that test-retest variability of layer thickness assessments is lower for this relatively large ROI compared to that of smaller ROIs [41]. Consequently, we reported on overall retinal changes. These results do not necessarily imply diffuse thinning, although they can be an outcome of diffuse changes or of multiple focal lesions, resulting in an average overall thinning.

The fovea has previously been a subject of investigation in *de novo* PD. One study reported a thinner central foveal thickness (mean (SD): PD = 240 (24.5) μm, HC = 255.3 (46) μm) [5]. The large variability of these measurements suggests a high amount of noise in measuring the foveal thickness, especially in HC. The macular GCL-IPL was also significantly thinner in PD compared to HC. This study confirms our findings of a significantly thinner macula in *de novo* PD. Further data was provided by a second study [4], where the fovea was not significantly thinner, thus contradicting the first study [5]. This second study further reported significant thinning of the GCL and the IPL in the inner temporal and inner inferior sectors [4]. The thinner inner temporal and inner inferior sectors are closest to the fovea but not part of the fovea. In conclusion, there is a limited amount of published data in the *de novo* PD literature regarding the fovea, which implies that further research is needed.

The retina has been studied in idiopathic rapid eye movement sleep behavior disorder (iRBD) patients, a prodromal PD population. One study reported a thinned pRNFL in iRBD patients [42]. Another study reported thinning in the GCC inner inferior, inner temporal, exterior nasal, and exterior temporal sectors [43]. The whole retinal thickness of the exterior temporal, inner superior, inner temporal, and inner inferior sectors were also thinner. Based on the raw data, the GCC thickness of iRBD patients appeared to be in between that of the HC and the PD patients for the inner superior and inner temporal sectors, suggesting that the thinning may be a mirror of the progression of PD. However, this was not statistically evaluated, and was not confirmed in the remaining sectors. A third study reported thinning of the RNFL, GCL, IPL, INL, OPL, and ONL in iRBD patients compared to HC, and also thinning in PD compared to HC [44]. The RNFL and GCL were thinner in PD versus iRBD, but none of the remaining layers were different. These results suggest a plausible progressive degeneration of the RNFL and the GCL as the disease progresses. These layers were not thinned in our data, suggesting that the IPL may be thinner already in the prodromal iRBD stage whereas the RNFL and GCL degenerate later. However, the study had a small sample size, and reported no correction for multiple comparisons [44]. Overall, there are few published studies including iRBD populations in retinal research and the results are not always consistent, which heralds the need for further research.

### Correlation analyses

The correlation analyses of the PD group did not show a correlation between the IPL thickness and the UPDRS III scores, after adjustment for age. The retinal cell layers of the macula are known to change with aging, so adjusting this data for age is necessary [45–47]. The lack of association between UPDRS III scores and retinal thicknesses (RNFL, GCIPL) has been previously reported in *de novo* PD patients, and also in cross-sectional data of non-*de novo* patients [5, 10, 48–50]. Two studies reported a negative correlation between retinal thickness and UPDRS III scores [51, 52]. The lack of correlation between IPL and UPDRS III at baseline might suggest that the IPL is not a good marker for the motor progression of PD, but follow-up data are needed to address this in more detail. We also could not find a correlation between IPL thickness and cognition. However, a few previous publications reported a significant correlation between the GCL-IPL and MoCA [53, 54]. One longitudinal study reported an increased risk of cognitive decline after three years of follow-up of PD patients with a lower RNFL and GCL-IPL thickness at baseline [53]. Furthermore, one study in *de novo* PD patients has reported a significant correlation between GCL-IPL thickness and MoCA (Pearson’s *r* = 0.471, *p* = <0.001, *n* = 74) [5]. We also did not find a significant association between MoCA and the joint GCL-IPL. A possible explanation for this discrepancy could be a difference in the MoCA score distribution of these studies, being 25.1 (2.9) in our cohort, compared to 23.9 (4.9) of the other *de novo* study, indicating a higher variability of MOCA scores in their study population. Previous work has shown that 29.8% of *de novo* PD patients from this cohort present with mild cognitive impairment, including memory and executive dysfunction, with good arguments showing a failing cholinergic upregulation, in the presence of cholinergic denervation [55]. As such, cognitive dysfunction is part of all stages of PD, including *de novo* PD, as shown in our cohort. Our study population presented with less variance in cognition, which could plausibly explain our results.

Data correlating visual function measures and retinal cell layer thicknesses have been previously reported [11, 50, 56, 57]. One longitudinal study reported a weak (*r* < 0.5) inverse correlation between the superotemporal RNFL and low contrast visual acuity, and also between the inferotemporal sector of the RNFL and visual acuity [11]. Another study reported weak correlations of the RNFL and the GCL-IPL with contrast sensitivity (static test), and also a weak correlation between contrast sensitivity (dynamic test), and the GCL-IPL [50]. This study also reported one strong correlation of macular thickness and contrast sensitivity (dynamic test) [50]. We could not replicate these findings, which could be due to the *de novo* disease stage of our patient population. Visual function test performance may be confounded by medication status, with improved or worsened visual performance [56]. Some studies reported strong correlations with subsectors of the ETDRS grid, but not with the average [11, 57]. This could possibly be an effect of multiple comparisons, and only some of the published results are corrected for this [11, 50]. Overall, there is a lack of sufficient published data to fully uncover the correlations between the retinal thicknesses and visual function in PD.

### Machine learning classification

Our machine learning classifier performed well. PD and POAG could be differentiated very well from each other, with excellent classification performance as measured by the ROC curve. PD and HC also could be differentiated very well by SD-OCT, but with a moderate classification performance. The machine learning analysis was carried out to evaluate the classification performance of individual patients between diagnostic categories of PD vs. HC, and PD vs. POAG, based on group level data. This allowed us to directly evaluate the possible diagnostic accuracy of SD-OCT in PD.

### Limitations and strengths

The most important strength of our study is the well-defined and strictly selected population, with a confirmed presynaptic dopaminergic deficiency, based on FDOPA-PET scanning. Another strength is the fact that the SD-OCT scans took place at an off-medication state. Therefore, the included PD- and POAG patients and HC were measured with the same methods, under the same circumstances. Another strength of this paper is the use of the Iowa reference algorithm, which is the only free available software able to segment the IPL. Only Spectralis (Heidelberg Engineering, Heidelberg, Germany) presents data from the GCL and IPL distinctly. Finally, we analyzed the macula and did not investigate the peripapillary RNFL, as the peripapillary RNFL thickness is more variable in PD and therefore has less predictive value, as shown in previous work [1]. A final strength of our analysis is the use of Bayesian informative hypotheses statistical analyses, which were additionally confirmed in post-hoc analysis with conventional *t*-tests.

A limitation of this study is the cross-sectional character of the dataset, which has shown some interesting differences in the thickness of particular retinal layers of PD- and POAG patients. However, only longitudinal OCT follow-up data will be able to identify potential biomarkers. We did not investigate focal areas of the retina (e.g., ETDRS grid sectors), or the foveal and parafoveal areas independently, and therefore our study cannot provide evidence for or against focal retinal thinning in *de novo* PD, which is a limitation. Our focus was on *de novo* PD patients, as such we cannot draw conclusions about advanced stages of PD. Furthermore, we reported that MoCA was not correlated with the IPL thickness, which might be due to the fact that MoCA is a screening instrument, lacking the depth of a detailed neuropsychological assessment.

### Future directions

SD-OCT should be complemented with functional testing of the visual system, like dynamic contrast sensitivity testing, a psychophysical approach targeting the amacrine cells [58]. A prodromal cohort of patients at risk for developing PD would contribute to the validation of SD-OCT as a possible diagnostic tool in PD. The influence of dopaminergic medication should be addressed by ophthalmic and SD-OCT measurements before and after administration of dopaminergic drugs.

### Conclusion

*De novo*, treatment-naive PD patients have a distinct retinal signature with reduction of especially the IPL thickness, compared to HC. The reduced thickness of the retina in *de novo* PD patients is also significantly different from early POAG patients, which suggests a possibly distinct pattern of retinal thinning in *de novo* PD patients. However, longitudinal OCT follow-up data and OCT data from other neurodegenerative diseases are needed to further analyze the sensitivity and specificity of OCT as a possible biomarker in PD patients.

## Data Availability

The data supporting the findings of this study are available on request from the corresponding author.
